# Seroprevalence of brucellosis in sheep and goats from Al Jufrah district in Libya

**DOI:** 10.11604/pamj.2024.48.23.38566

**Published:** 2024-05-28

**Authors:** Khawla Aldweni Alshekh, Aisha Mohamed Shahlol, Kholoud Khaled Ben Mostafa, Aeshah Abdulrrazaq Othman, Murad Ali Hiblu, Yousef Mohamed Abouzeed, Mohamed Ali Daw, Mohamed Omar Ahmed

**Affiliations:** 1 Department of Biological Sciences, School of Basic Sciences, The Libyan Academy of Graduate Studies, Tripoli, Libya,; 2 Department of Medical Lab Technology Science, University of Wadi-Al-Shatii, Brak, Libya,; 3 National Centre of Animal Health, Tripoli, Libya,; 4 Minister of Agriculture and Livestock, Tripoli, Libya,; 5 Department of Internal Medicine, Faculty of Veterinary Medicine, University of Tripoli, Tripoli, Libya,; 6 Department of Microbiology and Parasitology, Faculty of Veterinary Medicine, University of Tripoli, Tripoli, Libya,; 7 Department of Medical Microbiology, Faculty of Medicine, University of Tripoli, Tripoli, Libya

**Keywords:** Brucellosis, small ruminants, epidemiology, Al Jufrah district, Libya

## Abstract

**Introduction:**

brucellosis is a global neglected zoonotic disease affecting mainly livestock, causing communicable and zoonotic infections. This study aimed to investigate the seroprevalence and determine epidemiological risk factors associated with Brucella infection in sheep and goats in Al Jufrah central district of Libya.

**Methods:**

sera samples from 555 animals (goats (n=320) and sheep (n=235)) sheep) were obtained and subjected to the Rose Bengal Plate Test (RBPT) then further confirmed by a validated Enzyme Linked Immunosorbent assay (ELISA). Collected data was analyzed using Statistical Package for the Social Sciences (SPSS).

**Results:**

in total, 2.7% were ELISA seropositive for brucellosis with the highest seropositivity rate among the studied animals from Sokna with 5.8% (n=13/225) followed by 0.7% (n=2/285) in Waddan and 0% (n=0/45) in Houn. Only location was identified as a significant risk and no significant differences were identified between seropositivity and the age studied groups, species of animals, gender, and size of farms (p-value>0.05).

**Conclusion:**

the present study provides important information on the epidemiological status of Brucella infection in an important region in North Africa. Prevention control systems adopting “One Health” concept, and regional and international collaboration are important to control brucellosis and other zoonotic and transboundary diseases.

## Introduction

Brucellosis is a global neglected zoonotic disease affecting mainly livestock causing significant morbidity and economic losses [1,2]. *Brucella* consists of numerous species, and biovars exhibiting strong host specificity [3]. *A, Brucella abortus* (*B. abortus), Brucella ovis (B. ovis)* and *Brucella suis* (*B. suis)* are the most relevant species causing zoonotic infections and significant economic losses in livestock production, particularly in endemic underdeveloped regions [4,5]. For North African countries, investigations of brucellosis in small ruminants are limited, but available estimates include a prevalence range between 0.1% to 7.5% [6,7]. In these regions, brucellosis endemicity is mainly from *Brucella melitensis* (*B. melitensis)* mainly from goats and sheep followed by *B. abortus* isolated from natural and non-specific hosts with variable incidences and epidemiological distribution [8-13]. For instance, a nationwide study of brucellosis in the western mountain region of Libya covering the period between 2006-2008 reported a prevalence of 31% in goats, 42% in cattle, and 40% in humans with signs of active infections [14]. In Libya, the seroprevalence in humans suspected clinical samples in the northwestern region was highly reported, with an overall estimated incidence of 0.2-22 cases per 100,000 inhabitants [15]. In Eastern African countries, seroprevalence of brucellosis in small ruminants was reported to range from 0% to 20.0% among goats and 0% to 13.8% among sheep [16]. For the sub-Saharan region, about 16% of all livestock are estimated to be infected with brucellosis, mainly attributed to *B. abortus, B. melitensis* and *B. suis* with similar genetic lineages to the Mediterranean circulating lineages [5,9]. Although the Northern and sub-Saharan regions of Africa have substantial livestock populations and production systems, studies and epidemiological information on brucellosis from these regions are very scarce, including rural and coastal regions. Therefore, this study aimed to investigate the seroprevalence and epidemiological risk factors of *Brucella* infection in sheep and goats from October 2021 to February 2022 in Al Jufrah central district of Libya.

## Methods

**The study area:** Al Jufrah is one of the largest districts in the center of Libya, bordered by Surt to the north, Ajdabiya to the northeast, Al Kufra to the east, Murzuq to the south, Sabha to the southwest, Ash Shati to the west and Jabal al Gharbi to the northwest. The district occupied 52,342 square kilometers with an estimated population of 76,000 inhabitants. Sukna, Hun, and Waddan are the main town oases, followed by Zalla and Al fuckha. Animal husbandry and breeding in the district are mainly dependent on small ruminants with an estimation of 130,000 sheep and goats, whereas cattle and camels are the lowest (personal communications with local agriculture authorities).

**Criteria of Inclusion animals:** apparently healthy sheep and goats were included in the study based on the eligible criteria of non-clinical presentation of any illnesses except febrile at least six months before the study started. All included animals and premises were investigated for diseases and clinical manifestations related to abortion and diarrhoeal disease. Owners were given assurance of confidentiality and anonymity, and consents were obtained.

**Collection of data:** a semi-structured questionnaire was used to collect sociodemographic data and information on each flock´s animal characteristics, management status, and animal movements. Simple based “yes” or “no” questions were designed to acquire the relevant information consisting of sociodemographic data and selected variables related to potential risk factors for brucellosis infection and transmission between animals and from humans (i.e. age, species, location, gender, and size of premise). The questionnaire was designed and filled in English, but the interview was performed in Arabic language.

**Collection of blood samples:** a total volume of five milliliters (5 ml) of blood was collected from each animal by venipuncture in ethylenediaminetetraacetic acid (EDTA) tube and kept in a slanted position in cold icy boxes and then transported to the laboratory. Tubes were centrifuged at 5000 revolutions per minute (RPM) using a benchtop microprocessor-controlled centrifuge (HOSPITEC low-speed centrifuge model CH80-2) for 10-15 minutes. After centrifugation, the clear serum of each sample was transferred into sterile tubes, labelled and immediately stored at -20°C until further testing.

**Serological testing of samples:** the sera sample of animals were subjected to the Rose Bengal Plate Test (RBPT) and Enzyme-Linked Immunosorbent Assay (ELISA). *Brucella* antibodies testing using the RBPT was performed following the manufacturer guidelines (ID vet, Rose Bengal antigen for RSA test, ID vet 310, rue Louis Pasteur- Grabels- France). Indirect ELISA (iELISA) was done to further confirm RBPT-positive samples for agglutination against *Brucella* (IgG) antigen. The iELISA kit (ID vet France, kit reference BRUS-MS-5P) was used to test sera that characterize multispecies antibodies of smooth lipopolysaccharide (S-LPS) expressing *Brucella*, including *B. abortus, B. melitensis* and *B. suis*. The test plates were read using the ELISA reader (BioTek) at an optical density (OD) of 450 nm within 15 min.

**Statistical analysis:** the individual and flock-level prevalence was calculated by determining percentages and proportions of seropositive samples. All frequencies and potential risk factors for brucellosis were assessed for potential associated with seropositivity using a chi-square test at p≤0.05. P-values less than or equal to 0.05 were considered statistically significant. Data were analyzed using SPSS Statistics for Windows version 20 (SPSS-20, IBM Corp, Armonk, NY).

## Results

A total of 555 samples were collected originating from nine farms distributed between Waddan (n=285 samples; n=6 farms), Sokna (n=225 samples; n=2 farms) and Houn (n=45 samples; n=1 farms) representing 51.4%, 40.5% and 8.1% of the total collected samples, respectively. The age of animals ranged between 2-9 years (mean age 3.3 years). In total, 57.7% (n=320/555) were goats followed by 42.3% (n=235/555) sheep; 56.9% (n=316/555), further characterized as females of 56.9% (n=316/555), and males 43.1% (n=239/555). The nine included farms ranged between 3-35 acres and farm sizes >5 acres representing 85.8% (n=476/555) of the total samples, as well as farm size ≤5 acres representing 14.2% (n=79) (Table 1).

**Table 1:** number, proportion and seropositivity among sheep and goat flocks

Variables	Total collected number	ELISA Seropositive animals
**Area location**		
Waddan	N=285; 51.4%	N=2; 0.7%
Soukna	N=225; 40.5%	N=13; 5.8%
Houn	N=45; 8.1%	N=0; 0%
**Age**		
Less than 1 year	N=96; 17.3%	N=4; 4.2%
1-3 years	N=239; 43.1%	N=6; 2.5%
Over 3 years	N=220; 39.6%	N=5; 2.3%
**Species**		
Sheep	N=235; 42.3%	N=9; 3.8%
Goats	N=320; 57.7%	N=6; 1.9%
**Gender**		
Male	N=239; 43.1%	N=5; 2.1%
Females	N=316; 56.9%	N=10; 3.2%
**Size farm (acres)**		
≤ 5 acres	N=79; 14.2%	N=0; 0%
> 5 acres	N=476; 85.8%	N=15; 3.2%

**ELISA:** enzyme-linked immunosorbent assay

**Seropositivity among sheep and goat flocks:** in total, 2.7% of samples were ELISA seropositive for brucellosis with the highest seropositivity rate from Sokna at 5.8% (n=13/225) followed by Waddan at 0.7% (n=2/285) and Houn at 0% (n=0/45). A higher seropositivity was found in sheep at 3.8% (n=9/335) and only 1.9% (n=6/320) in goats. Females had a higher seropositivity rate, representing 3.2% (n=10/316) compared to 2.1% (n=5/239) among males. In addition, the age group of less than one year old had higher seropositivity at 4.2% (n=4/96) followed by the age group of 1-3 years with 2.5% (n=6/239) seropositivity, then the group over 3 years with the least seropositivity at 2.3% (n=5/220). Seropositive samples all originated from a farm size of >5 acres (n=15) (Table 1).

**Association between seropositivity and selected studied variables:** there were no significant differences or associations between the studied variables and seropositivity, except for the location the samples were obtained. There was a significant difference between the three studied locations (P=0.01, p-value<0.05); however, 86.7% (n=13/15) of cases were from Soukna followed by Waddan at 13.3% (n=2/15) and no case was reported from Houn. Furthermore, no significant differences between the age studied groups and seropositivity (P=0.61), with higher seropositivity in the 1-3 years age group at 40% followed by age group > 3 years at 26.7%, whereas the age group < 1 year was the lowest. There were no significant differences between animal species (P=0.18, p-value>0.05); however, the highest seropositivity was among sheep at 60% then goats at 40%. There were no significant differences (P=0.42) in seropositivity between females at 66.7% and males at 33.3%. Also, no significant differences were found regarding the two categorized farms' size (P=0.09); nevertheless, all positive samples (n=15) originated from farm sizes of >5 acres (Table 2).

**Table 2:** association between seropositivity and selected studied variables

Variables	Number & proportion of samples		Chi-square test	Probability (P) value	Correlation coefficient value
	Sero- (+)	Sero- (-)			
**Location**			13.68	0.01	0.15
Waddan (n=285)	13.3 (2)	52.4 (283)			
Houn (n=45)	0(0)	8.3 (45)			
Soukna (n=225)	86.7 (13)	39.3 (212)			
**Age**			0.97	0.61	0.04
< 1 year (n=96)	4 (26.7%)	92 (17%)			
1-3 years (n=239)	6 (40%)	233 (43.1%)			
> 3 years (n=220)	5 (33.3%)	215 (39.8%)			
**Animal species**			1.96	0.18	0.23
Sheep (n=235)	60% (9)	58.1% (225)			
Goats (n=320)	40% (6)	41.9% (314)			
**Animal gender**			0.59	0.42	-0.26
Female (n=316)	66.7% (10)	56.7% (306)			
Male (n=239)	33.3% (5)	43.3% (234)			
**Farm Size**			2.55	0.09	0.06
≤ 5 acres (n=79)	0 (0)	14.6 (79)			
> 5 acres (n=476)	100 (15)	85.4 (461)			

**Abbreviations:** Sero- (+), Seropositive, Sero- (+), Seronegative

## Discussion

Brucellosis is a major endemic disease of small ruminants, particularly in North African countries [17]. Human brucellosis in this region was reported in the 1980s however, the epidemiological distribution of brucellosis in this endemic region is not fully elucidated and the real incidence of notifiable infections in some regions might be highly underestimated due to different and complicating regional factors [9,18]. The overall estimated prevalence in the present study was 2.7%, where only location was identified as the significant risk among the three studied locations, with Soukna as the highest seropositivity rate at 5.8%. Waddan, Hun and Soukna are respectively the main populated town oases dependent mainly on sheep and goats raised in large premises located in the outskirt areas. Soukna has fewer farms and livestock population compared to Waddan; however, Soukna has larger extensive breeding and concentration of animals within their farms, which may contribute to the higher rate of brucellosis infection.

Generally, the brucellosis prevalence in this study was lower than previously reported from the North-West region of Libya, which might be attributed to the particular location and environmental features of the district in the central region of the country bordering the desert and Sahar. Both port and coastal routes located in the Northern coastal regions are the main introducing route and export of animals destined for human consumption in the local markets, thus playing an important role in the introduction and spread of communicable diseases. In addition, exported animals from neighbouring sub-Saharan African countries and Sudan usually pass through this area towards other Libyan regions and such animal mobility is not entirely controlled due to the instability of the region and the potential illegal trade of animals.

Brucellae is a major problem for ruminants and dairy animals in Africa and is highly contagious, particularly through direct contact with infected animals or materials [19,20]. Humans are also infected via the consumption of unpasteurized milk and dairy products, as well as undercooked meat [21-23]. *Brucella* may also be transmitted to humans via skin abrasion, conjunctival inoculation, inhalation of contaminated aerosol, blood transfusion, transplantation, and vertical transmission via breastfeeding [1,24]. Brucellae is also an important occupational hazard that can infect slaughterhouse workers, veterinarians, and farmers, causing serious zoonotic, clinical, and chronic infections [2]. Also, relapsing after therapy may develop with brucellosis mainly due to acquired or intrinsic resistance against antimicrobial drugs [25,26].

In North Africa, *Brucella* species from animals were reported with evolved genetic properties suggesting the continuous evolution of brucellosis, attributed mainly to the mixed breeding systems [27]. For instance, a recent study performed in Egypt´s Delta region detected *Brucella* seropositivity in sheep between 6.7-7.2% and identified *B. melitensis* (biovar 3) as the prevalent serotype [28]. Also, *Brucella*-causing human infections caused by lineages of *B. melitensis* biovar were reported to be potentially associated with socio-historical connections with Europe [9]. Unfortunately, such molecular information is not available from many North African regions, particularly Libya, which is attributed to the lack of laboratory and diagnostic infrastructures.

In Libya, brucellosis is an important communicable disease and a significant public health concern, mainly in the northwest region. Diagnostic testing and treatment of brucellosis are maintained by 28 healthcare facilities distributed in five districts located entirely in the highly endemic northwest region of the country, except one facility located in Sebha in the southern region [29]. These facilities have limited capacity for medical services to diagnose brucellosis in humans, depending on non-laboratory methods such as clinical history and common laboratory serological tests. Similarly, diagnostic testing for animals is inadequate and lacks adequate laboratory infrastructures and trained personnel and is mostly performed in the northwest region depending on clinical and serological tests.

In North African countries including Algeria, Morocco, and Tunisia, prevention and control measures against brucellosis in small ruminants are variably adopted, such as mass vaccination and/or testing and slaughter of infected animals [10]. However, the transmission of brucellosis was not efficiently controlled mainly due to local and cultural factors such as consumption of raw milk and milk products and/or inadequate prevention practices [14,30]. In contrast, brucellosis was controlled in cattle in the North region of the Mediterranean region, mainly due to the banning of importation of animals from the North African endemic areas [5].

International guidelines for countries affected by brucellosis in animals are recommended to control and diagnose guidelines including serology, slaughtering of seropositive animals, vaccination programmes, and strict hygienic measures [31]. For instance, the iELISA-based kit that was used in the current study to test sera for multispecies antibodies of smooth lipopolysaccharide (S-LPS) expressing *B. abortus, B. melitensis*, and *B. suis* is the widely adopted test to screen for brucellosis showing high sensitivity and specificity at 96.8% and 96.3%, respectively [31,32]. Also, there is no active national prevention scheme or system for brucellosis in animals in Libya. The current political instability in the North African region, particularly Libya has certainly impacted any ongoing plans to contain any spread and transmission.

## Conclusion

The present study provides essential knowledge and information on the epidemiological status of *Brucella* infection in this North African region. Controlling and management of brucellosis in livestock is required, and adequate laboratory and diagnostic infrastructures are necessary. Prevention control systems adopting “One Health” concept, and regional and international collaboration are important to control brucellosis and other zoonotic and transboundary diseases.

### What is known about this topic

- *Brucellosis is a global zoonotic and neglected disease with major public health importance;*
- *Brucellosis is endemic in many African countries, particularly the sub-Saharan region;*
- *Serological methodologies are essential laboratory and epidemiological tools for Brucella infections and brucellosis including RBPT and indirect ELISA techniques, particularly in developing and low-economic countries*.

### What this study adds

- *This study confirmed the presence and the seroprevalence of Brucella infection in small ruminant animals in important sub-Saharan regions in Africa;*
- *This study provides novel data and showed a moderate seroprevalence rate of Brucella infections using RBPT, and indirect ELISA among goats and sheep;*
- *The study identifies and discusses risk and regional factors associated with Brucella infection at the regional level*.
